# Decreased COPD prevalence in Sweden after decades of decrease in smoking

**DOI:** 10.1186/s12931-020-01536-4

**Published:** 2020-10-28

**Authors:** Helena Backman, Lowie Vanfleteren, Anne Lindberg, Linda Ekerljung, Caroline Stridsman, Malin Axelsson, Ulf Nilsson, Bright I. Nwaru, Sami Sawalha, Berne Eriksson, Linnea Hedman, Madeleine Rådinger, Sven-Arne Jansson, Anders Ullman, Hannu Kankaanranta, Jan Lötvall, Eva Rönmark, Bo Lundbäck

**Affiliations:** 1 Department of Public Health and Clinical Medicine, Section of Sustainable Health/the OLIN Unit, Umeå University, Umeå, Sweden; 2 COPD Center, Sahlgrenska University Hospital, University of Gothenburg, Göteborg, Sweden; 3 Dept of Public Health and Clinical Medicine, Section of Medicine, Umeå University, Umeå, Sweden; 4 Krefting Research Centre, Institute of Medicine, University of Gothenburg, Göteborg, Sweden; 5 Department of Care Science, Faculty of Health and Society, Malmö University, Malmö, Sweden; 6 Dept of Health Sciences, Luleå University of Technology, Luleå, Sweden; 7 Department of Medicine, Halmstad Central County Hospital, Halmstad, Sweden; 8 Wallenberg Centre for Molecular and Translational Medicine, Institute of Medicine, University of Gothenburg, Göteborg, Sweden; 9 Department of Respiratory Medicine, Seinäjoki Central Hospital, Seinäjoki, Finland; 10 Faculty of Medicine and Health Technology, Tampere University, Tampere, Finland

**Keywords:** COPD, Prevalence, Risk, Population study, Epidemiology

## Abstract

**Background:**

COPD has increased in prevalence worldwide over several decades until the first decade after the millennium shift. Evidence from a few recent population studies indicate that the prevalence may be levelling or even decreasing in some areas in Europe. Since the 1970s, a substantial and ongoing decrease in smoking prevalence has been observed in several European countries including Sweden. The aim of the current study was to estimate the prevalence, characteristics and risk factors for COPD in the Swedish general population. A further aim was to estimate the prevalence trend of COPD in Northern Sweden from 1994 to 2009.

**Methods:**

Two large random population samples were invited to spirometry with bronchodilator testing and structured interviews in 2009–2012, one in south-western and one in northern Sweden, n = 1839 participants in total. The results from northern Sweden were compared to a study performed 15 years earlier in the same area and age-span. The diagnosis of COPD required both chronic airway obstruction (CAO) and the presence of respiratory symptoms, in line with the GOLD documents since 2017. CAO was defined as post-bronchodilator FEV_1_/FVC < 0.70, with sensitivity analyses based on the FEV_1_/FVC < lower limit of normal (LLN) criterion.

**Results:**

Based on the fixed ratio definition, the prevalence of COPD was 7.0% (men 8.3%; women 5.8%) in 2009–2012. The prevalence of moderate to severe (GOLD ≥ 2) COPD was 3.5%. The LLN based results were about 30% lower. Smoking, occupational exposures, and older age were risk factors for COPD, whereof smoking was the most dominating risk factor. In northern Sweden the prevalence of COPD, particularly moderate to severe COPD, decreased significantly from 1994 to 2009, and the decrease followed a decrease in smoking.

**Conclusions:**

The prevalence of COPD has decreased in Sweden, and the prevalence of moderate to severe COPD was particularly low. The decrease follows a major decrease in smoking prevalence over several decades, but smoking remained the dominating risk factor for COPD.

## Background

Chronic obstructive pulmonary disease (COPD) is a common disease worldwide and a major public health problem. Globally, the prevalence of COPD has increased during the past century with tobacco smoking being the most important risk factor especially in high income countries [1]. Epidemiological data have indicated that up to 50% of smokers may develop COPD sooner or later if they continue to smoke [2, 3], and the incidence of COPD is high among smokers already at younger ages [4, 5].

Since the 1970s, a substantial and ongoing decrease in smoking prevalence has been observed particularly in North America, Australia, and in several northern and western European countries [6], at least partly due to different public health interventions. Positively, some recent studies based on spirometry have indicated the prevalence trend of COPD to be levelling off or even tending to decrease [7–10]. The substantial reduction in smoking will probably contribute to a reduced future burden of COPD.

Along with spirometric classification of chronic airway obstruction and risk of exacerbations, the recent GOLD strategy documents also emphasize the importance of assessing respiratory symptoms in the diagnosis and management of COPD [11]. However, the knowledge on prevalence trends and risk factors for COPD based on both airway obstruction confirmed by spirometry and respiratory symptoms is limited.

The aim of the current study was to estimate the prevalence, characteristics and risk factors for COPD in the Swedish general population using clinical and physiological methods according to the GOLD 2020 document [11], by using also the LLN-criterion. A further aim was to estimate the prevalence trend of COPD in Northern Sweden from 1994 to 2009. We hypothesized that the prevalence of COPD had decreased and that the risk factor pattern was altered following the major decrease in smoking in Sweden.

## Methods

The study was performed in Norrbotten in northern Sweden and in Västra Götaland in south-western Sweden within two large scale population-based research projects, the Obstructive Lung Disease in Northern Sweden (OLIN) Studies, and the West Sweden Asthma Study (WSAS). The study comprises clinical examinations performed in 2009–2012 [12] including structured interviews and spirometry with reversibility testing in the age-range 21–78 years. The Masterscope (Jaeger) spirometer was used in both study areas. Approval was received from the Regional Ethical Review Boards at the Universities of Umeå and Gothenburg, Sweden. All participants signed informed consent.

The study population (Additional file 1: Fig. S1) included 1839 randomly selected responders from two large scale questionnaire surveys, which well reflected the age and gender distribution of the population in the two areas. Furthermore, the results from northern Sweden were also compared with a previous methodologically similar study in 1994 (n = 660) in the same area and using same age-span [9]. Methods are described in more detail in the e-Appendix.

### Definitions

*COPD*: Both chronic airway obstruction (CAO) and the presence of respiratory symptoms were required, in line with GOLD 2020 [11]. In the main analyses, *CAO* was defined as post-bronchodilator FEV_1_/FVC < 0.7 (the fixed ratio criterion). Results based on the FEV_1_/FVC < lower limit of normal (LLN) criterion are given as on-line materials for comparison. Swedish reference values for spirometry were used [13]. The symptoms required for the diagnosis of COPD included at least one of the following respiratory symptoms: longstanding cough, chronic productive cough, sputum production, mMRC dyspnea scale ≥ 2, recurrent wheeze, persistent wheeze and/or attacks of shortness of breath, all chronic or recurrent within the last 12 months. Furthermore, based on information on exacerbations and mMRC dyspnea scale (range 0–4) collected at the structured interviews, COPD was divided into the GOLD categories A, B, C and D [11]. *Exacerbations* were defined as a worsening of respiratory symptoms last 12 months leading to hospitalization, other health care contacts, or use of antibiotics or oral corticosteroids. *Moderate to severe COPD* was defined following GOLD ≥ 2 (FEV1 < 80% of predicted) in combination with respiratory symptoms.

Based on detailed information collected at the structured interview, *smoking* was categorized both by packyears and current smoking status: never-smokers, ex-smokers (having quit since at least 12 months) or current smokers. Ever heavy *exposure to gas, dust or fumes (GDF) at work* was assessed [12]. *BMI* was categorized as Underweight (BMI < 20), Normal weight (20 ≤ BMI < 25), Overweight (25 ≤ BMI < 30) and Obesity (BMI ≥ 30). Socioeconomic status was based on *educational level*.

### Statistical analyses

The IBM SPSS Statistics (IBM Corp. Released 2017. IBM SPSS Statistics for Windows, Version 25.0. Armonk, NY: IBM Corp.) was used for statistical analyses. In bivariate analyses, the Chi-square test was used to test for differences in proportions and ANOVA for differences in means. P-values < 0.05 from two-tailed tests were considered statistically significant. Risk factors for CAO and COPD were analyzed by logistic regression with results expressed as odds ratios (OR) with 95% confidence intervals (CI). The combined variable method based on mutually exclusive categories was used for assessment of interaction between respectively smoking and occupational exposure to GDF, and smoking habits and sex, adjusted for age, educational level, exposure to GDF and family history of asthma by logistic regression. Results from 1994 were not available from south-western Sweden, why trends in COPD prevalence were studied based on the 1994 and 2009-samples from northern Sweden, analyzed by Poisson regression with results expressed as prevalence ratios (PR) with 95% CI.

Analyses utilizing the LLN-definition of CAO, including the Global Lung function Initiative LLN-definition [14], were performed to enable comparisons with other studies. These are briefly presented in the results section, with more details provided in the online materials.

## Results

### Basic characteristics

In 2009–2012, the mean age was 51.1 ± 14.8SD years, 52.6% were women and 12.8% were current smokers. The mean BMI was higher and exposure to gas, dust or fumes at work more common among men, while university education was more common among women (Table 1).

**Table 1:** Basic characteristics of the study sample examined during 2009–2012

Characteristic		Sex		Area		All	P-value for difference by	
		Women	Men	South-western Sweden	Northern Sweden			
		n = 968	n = 871	n = 1148	n = 691	n = 1839	Sex	Area
Age	Mean (SD)	50.3 (14.9)	52.1 (14.8)	50.6 (15.1)	52.0 (14.3)	51.1 (14.8)	**0.010**	0.058
BMI	Mean (SD)	26.3 (4.8)	26.9 (3.7)	26.2 (4.2)	27.2 (4.5)	26.6 (4.4)	**0.003**	** < 0.001**
Never-smoker	n (%)	491 (50.7%)	475 (54.6%)	610 (53.1%)	356 (51.6%)	966 (52.6%)		
Ex-smoker	n (%)	324 (33.5%)	313 (36.0%)	407 (35.5%)	230 (33.3%)	637 (34.7%)		
Current smoker	n (%)	153 (15.8%)	82 (9.4%)	131 (11.4%)	104 (15.1%)	235 (12.8%)	** < 0.001**	0.072
Never-smoker	n (%)	491 (51.3%)	475 (54.8%)	610 (53.5%)	356 (52.0%)	966 (53.0%)		
≤ 10 packyears	n (%)	244 (25.5%)	189 (21.8%)	266 (23.3%)	167 (24.4%)	433 (23.7%)		
11–20 packyears	n (%)	126 (13.2%)	61 (7.0%)	114 (10.0%)	73 (10.7%)	187 (10.3%)		
21–30 packyears	n (%)	64 (6.7%)	61 (7.0%)	71 (6.2%)	54 (7.9%)	125 (6.9%)		
> 30 packyears	n (%)	33 (3.4%)	80 (9.2%)	79 (6.9%)	34 (5.0%)	113 (6.2%)	** < 0.001**	0.292
Exp to GDF at work	n (%)	142 (14.8%)	353 (41.1%)	282 (24.7%)	213 (31.6%)	495 (27.3%)	** < 0.001**	**0.002**
University education	n (%)	447 (46.7%)	303 (35.5%)	533 (46.6%)	217 (32.6%)	750 (41.4%)	** < 0.001**	** < 0.001**

*BMI* Body Mass Index. One subject from the OLIN sample lack information on smoking habits. Information on number of packyears is lacking for 6 ever-smokers from OLIN and 8 ever-smokers from WSAS. Information on Exp to GDF at work is lacking for 16 subjects from OLIN and 8 subjects from WSAS. Information on Educational level is lacking for 25 subjects from OLIN and 3 subjects from WSAS. P-values from pearson's chi-square or student's T-test, as appropriate

Bold font indicates P < 0.05

Smoking habits differed by sex, where 15.8% of the women were current smokers compared to 9.4% of the men. In contrast, 9.2% of the men had a smoking history of > 30 packyears compared to 3.4% of the women (Table 1). Among current smokers, men had more packyears than women (mean 24.4 vs 17.6, P = 0.001). Additionally, when taking both current smoking and exposure to GDF into account, 26.7% of the women were exposed compared to 45.1% of the men (P < 0.001).

### Prevalence of CAO and COPD in 2009–2012

The prevalence (95% CI) of CAO was overall 8.7% (7.4–10.0). This was 7.5% (5.9–9.2) among women and 10.0% (8.0–12.0) among men (P = 0.063). The prevalence of COPD was overall 7.0% (5.8–8.1). This was 5.8% (4.6–7.0) among women and 8.3% (6.4–10.0) among men (P = 0.037). The prevalence of moderate to severe CAO and COPD was 3.9% and 3.5%, respectively, and both were more common among men. The prevalence did not differ between south-western and northern Sweden (Table 2). In ages ≥ 40 years (n = 1380), the prevalence of CAO was 10.7% (9.0–12.3); 9.6% (7.4–11.7) among women and 11.8% (9.4–14.3) among men (P = 0.177). The prevalence of COPD was 8.6% (7.1–10.0); 7.3% (5.4–9.2) among women compared to 9.9% (7.6–12.1) among men (P = 0.090). In ages ≥ 60 years (n = 586), the prevalence of CAO was 16.7% (13.7–19.7); 16.5% (12.1–20.9) among women and 16.9% (12.8–21.1) among men. The prevalence of COPD was 12.8% (10.1–15.5); 12.5% (8.5–16.4) among women compared to 13.1% (9.4–16.8) among men.

**Table 2:** Prevalence (n (%)) of COPD and CAO by age groups, sex, area and among all subjects

	Age groups						Sex		Area		All
	< 40 years		41–60 years		> 60 years						
	Women	Men	Women	Men	Women	Men	Women	Men	Western Sweden	Northern Sweden	
	n = 257	n = 202	n = 438	n = 356	n = 273	n = 313	n = 968	n = 871	n = 1148	n = 691	n = 1839
CAO: FEV1/FVC < 0.7	5 (1.9)	8 (4.0)	23 (5.3)	26 (7.3)	45 (16.5)	53 (16.9)	73 (7.5)	87 (10.0)	93 (8.4)	64 (9.3)	160 (8.7)
CAO: FEV1/FVC < 0.7 & FEV1 < 80%	1 (0.4)	1 (0.5)	6 (1.4)	15 (4.2)	23 (8.4)	26 (8.3)	30 (3.1)	42 (4.8)	42 (3.7)	30 (4.3)	72 (3.9)
COPD: FEV1/FVC < 0.7	4 (1.6)	6 (3.0)	18 (4.1)	25 (7.0)	34 (12.5)	41 (13.1)	56 (5.8)	73 (8.3)	74 (6.4)	54 (7.8)	128 (7.0)
COPD: FEV1/FVC < 0.7 & FEV1 < 80%	1 (0.4)	1 (0.5)	5 (1.1)	15 (4.2)	19 (7.0)	24 (7.7)	25 (2.6)	40 (4.6)	38 (3.3)	27 (3.9)	65 (3.5)

*CAO* Post-bronchodilator chronic airway obstruction, *COPD* CAO in combination with respiratory symptoms

Prevalence is reported as n (%). COPD: FEV1/FVC < 0.7 & FEV1 < 80% of predicted corresponds to moderate to severe COPD, i.e. GOLD stage ≥ 2

**Fig. 1:**
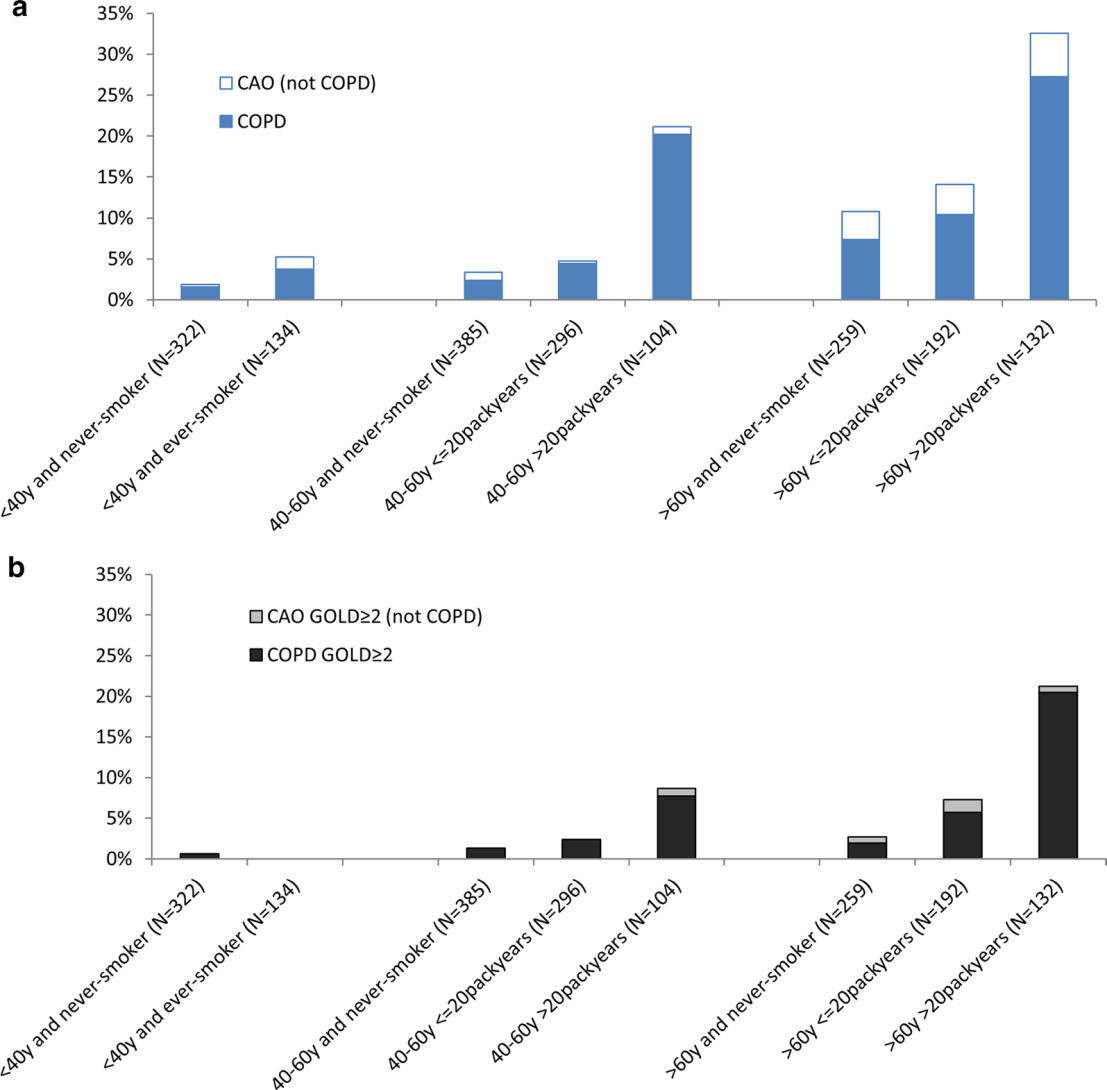
Prevalence of **a** CAO and COPD and **b** CAO GOLD ≥ 2 and COPD GOLD ≥ 2 by age and packyears of smoking. CAO = post-BD FEV_1_/FVC < 0.7, COPD = CAO in combination with respiratory symptoms

The prevalence of both CAO and COPD increased considerably by increasing age and number of packyears, particularly of moderate to severe COPD. Further, subjects with CAO but without respiratory symptoms were mainly aged > 60 years (Fig. 1a and b).

Among subjects with COPD, 27% reported exacerbations, while 67% reported neither exacerbations nor mMRC ≥ 2 (Additional file 2: Fig. S2). The prevalence of multimorbidity was considerably higher in subjects with than without COPD (Additional file 3: Table S1).

### Risk factors for CAO and COPD in 2009–2012

**Fig. 2:**
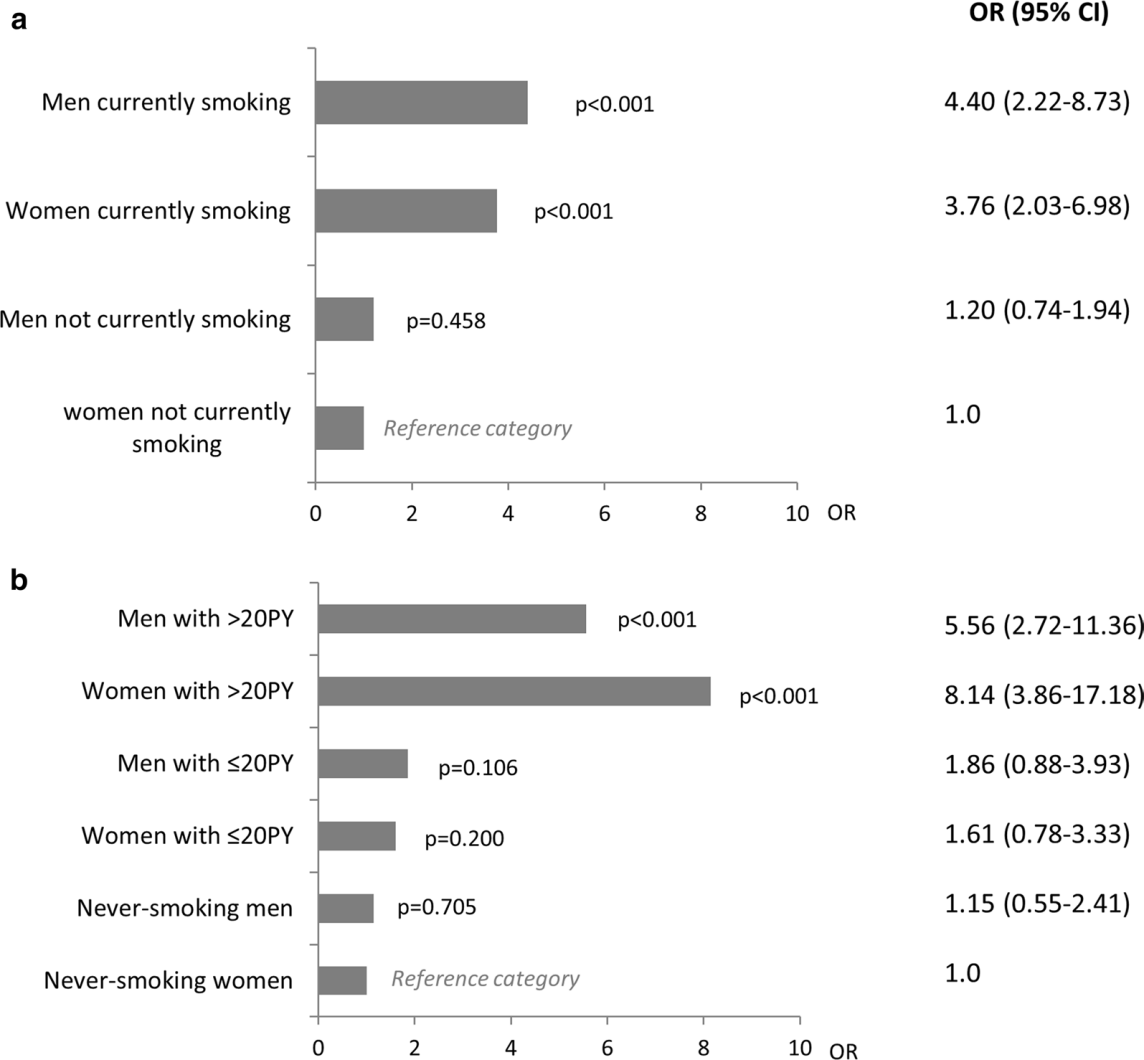
Interaction analyses for the risk of COPD and **a** smoking habits and sex, and **b** packyears of smoking and sex. Results expressed as Odds Ratios (OR) with 95% Confidence Intervals (CI) adjusted for age, level of education, age category, exposure to gas, dust or fumes at work and family history of obstructive airway disease. COPD was defined as post-BD FEV_1_/FVC < 0.7 in combination with respiratory symptoms. P-values for comparison with reference category

Results of unadjusted analyses are displayed in Additional file 3: Table S2. According to the adjusted analyses, age > 60 years was a significant risk factor (OR (95% CI)) for both CAO (OR 4.1, (2.1–7.8)) and COPD (OR 3.71.8–7.9). Having more than 10 packyears of smoking was strongly associated with both CAO and COPD, with the highest risk for > 30 packyears (OR 6.5 (3.8–11.1) for CAO, OR 7.5 (4.2–13.4) for COPD). Exposure to GDF at work was significantly associated with COPD (OR 1.5 (1.01–2.4)). Neither male sex nor lack of university education were significant risk factors for CAO or COPD (Table 3). The interaction analyses between sex and smoking revealed that the OR for COPD was 4.4 (2.2–8.7) among currently smoking men compared to 3.8 (2.0–7.0) among currently smoking women (Fig. 2a), and further that the OR for COPD was 8.1 (3.9–17.2) among women with a smoking history of > 20 packyears compared to 5.6 (2.7–11.4) among men (Fig. 2b). Interaction analyses between smoking and exposure to GDF at work indicated additive effects and confirmed smoking as the main risk factor for COPD (Additional file 4: Fig. S3).

**Table 3:** Risk factors for chronic airway obstruction (CAO) and COPD

Covariate	CAO		COPD	
	OR	(95% CI)	OR	(95% CI)
< 40 years	Reference		Reference	
40–60 years	1.51	(0.77–2.94)	1.68	(0.79–3.58)
> 60 years	**4.08**	**(2.13–7.81)**	**3.73**	**(1.77–7.87)**
Male sex	0.98	(0.67–1.43)	0.97	(0.64–1.46)
University education	Reference		Reference	
Non-university edu	1.07	(0.72–1.59)	1.02	(0.66–1.57)
Never-smoker	Reference		Reference	
≤ 10 packyears	1.18	(0.70–1.98)	1.28	(0.71–2.29)
11–20 packyears	**2.37**	**(1.37–4.11)**	**2.51**	**(1.35–4.67)**
21–30 packyears	**3.98**	**(2.26–7.01)**	**5.01**	**(2.73–9.19)**
> 30 packyears	**6.53**	**(3.84–11.10)**	**7.54**	**(4.24–13.42)**
Exp to GDF at work	1.36	(0.91–2.01)	**1.50**	**(1.01–2.36)**

Results presented as Odds Ratios (OR) with 95% Confidence Intervals (CI) from multiple logistic regression analyses

Binomial logistic regression for COPD vs non-COPD and CAO vs non-CAO, respectively

CAO = Post-bronchodilator chronic airway obstruction according to the fixed ratio definition (FEV1/FVC < 0.7)

COPD = CAO in combination with respiratory symptoms. GDF = Gas, dust or fumes

BMI-categories did not yield significant associations and were not included

Bold font indicates P < 0.05

### COPD prevalence change in northern Sweden from 1994 to 2009

In the 1994 northern Sweden OLIN cohort (mean age 49.1, mean BMI 25.7, 26.6% current smokers) the prevalence of COPD was 9.2% (Additional file 3: Table S3). Table 4 summarizes the prevalence differences in terms of prevalence ratios (PR) comparing 2009 with 1994 and shows a decrease in COPD prevalence with 41% (PR 0.59, 95% CI 0.39–0.89), together with a decrease in current smoking to 16.1% (P < 0.001). Adjusted for age and sex by Poisson regression, the prevalence decreased by 44% (PR 0.56, 95% CI 0.38–0.83). When adjusted for age, sex, BMI and socioeconomic status, the decrease in prevalence was 38% (PR 0.62, 95% CI 0.42–0.93), but when further adjusting also for smoking the significance was lost. The prevalence of COPD decreased by 50% (P = 0.033) among women and by 34% (P = 0.127) among men. The corresponding age-adjusted prevalence decrease was 53% (P = 0.019) among women and 36% (P = 0.085) among men, and the decrease among women remained significant also when adjusted for age, sex, BMI, socioeconomic status and smoking (Table 4). Among subjects with COPD in 1994 compared to 2009, the mean age was 54.1y compared to 59.5y (P = 0.025), the proportion of never-smokers 18.0% compared to 14.7% (P = 0.678), and the proportion with any physician-diagnosed obstructive airway disease was 14.8% compared to 29.4% (P = 0.087). When limiting the samples to ages > 40y (n = 946), the prevalence of COPD decreased by 35% (PR 0.65, 95% CI 0.43–0.99). When adjusted for age, sex, smoking, BMI, and socioeconomic status, the decrease in COPD prevalence in ages > 40y was no longer significant (P = 0.215, Table 4).

**Table 4:** Comparison between prevalence of COPD and moderate to severe COPD (GOLD ≥ 2) in 2009 and 1994 in Northern Sweden

	Unadjusted			Adjusted for age and sex			Fully adjusted		
	PR	95% CI	P-value	PR	95% CI	P-value	PR	95% CI	P-value
All subjects									
COPD	0.59	(0.39–0.89)	**0.011**	0.56	(0.38–0.83)	**0.004**	0.69	(0.46–1.03)	0.071
GOLD ≥ 2	0.40	(0.24–0.66)	** < 0.001**	0.38	(0.23–0.62)	** < 0.001**	0.47	(0.28–0.77)	**0.003**
Women									
COPD	0.50	(0.26–0.95)	**0.033**	0.47	(0.25–0.88)	**0.019**	0.51	(0.27–0.97)	**0.040**
GOLD ≥ 2	0.36	(0.15–0.83)	**0.017**	0.33	(0.15–0.76)	**0.009**	0.33	(0.14–0.78)	**0.012**
Men									
COPD	0.66	(0.39–1.12)	0.127	0.64	(0.38–1.06)	0.085	0.84	(0.51–1.38)	0.489
GOLD ≥ 2	0.42	(0.23–0.79)	**0.007**	0.41	(0.22–0.75)	**0.004**	0.56	(0.30–1.02)	0.059
Subjects aged > 40 years									
COPD	0.65	(0.43–0.99)	**0.043**	0.63	(0.42–0.95)	**0.027**	0.77	(0.50–1.17)	0.215
GOLD ≥ 2	0.44	(0.26–0.73)	**0.002**	0.42	(0.26–0.70)	**0.001**	0.52	(0.31–0.86)	**0.011**

Results expressed as prevalence ratios (PR) with 95% CI from Poisson regression analyses, comparing 2009 with 1994

PR = Prevalence ratio comparing the prevalence in 2009 with the prevalence in 1994

The fully adjusted model includes year of study, age, sex, BMI categories, socioeconomy, and smoking habits as covariates. P-values from Wald chi-square test. Information on exposure to gas, dust or fumes not available in 1994 and thus not included in the models. Bold font indicates P < 0.05

COPD = Post-bronchodilator chronic airway obstruction according to the fixed ratio definition (FEV1/FVC < 0.7) in combination with respiratory symptoms. GOLD ≥ 2 = COPD with post-BD FEV1 < 80% of predicted

### Prevalence change of moderate to severe COPD in Northern Sweden from 1994 to 2009

The prevalence of moderate to severe COPD (GOLD ≥ 2) decreased by 60% (PR 0.40, 95% CI 0.24–0.66) from 1994 to 2009 (Table 4). Adjusted for age and sex by Poisson regression, the prevalence decreased by 62%, and the decrease remained significant also when adjusted for age, sex, BMI categories, socioeconomic status and smoking. The decrease in moderate to severe COPD was more obvious among women than among men. When considering the subjects with moderate to severe COPD in the two surveys, the mean age increased from 55.4 to 61.2 years (P = 0.036), and the proportion with any physician-diagnosed obstructive airway disease increased from 15.1% to 40.0% (P = 0.022). When limiting the samples to ages > 40 years, the prevalence of moderate to severe COPD (GOLD ≥ 2) decreased by 56% (PR 0.44, 95% CI 0.26–0.73), and this decrease remained significant also when adjusted for age, sex, smoking, BMI, and socioeconomic status (Table 4).

### Lower limit of normal definition for COPD

Overall, the LLN-based results on COPD prevalence were about 30% lower than the fixed-ratio based results. The COPD cases among the young people fulfilling the COPD criterion with the LLN-definition but not with the fixed ratio definition were only few (n = 2 in ages ≤ 40 years). Further, when applying the LLN criterion of COPD among all subjects (regardless of age), n = 13 (20%) out of the 65 subjects with GOLD ≥ 2 according to the fixed ratio criterion for COPD were classified as non-COPD. The significant decrease in COPD prevalence from 1994 to 2009 in Northern Sweden was confirmed also based on the LLN-definition (Additional file 5: Appendix S1 and Additional file 3: Tables S4–S6). A further result was that the LLN-based findings indicated an interaction between smoking and GDF exposure on the risk for COPD (Additional file 4: Fig. S3).

## Discussion

**Fig. 3:**
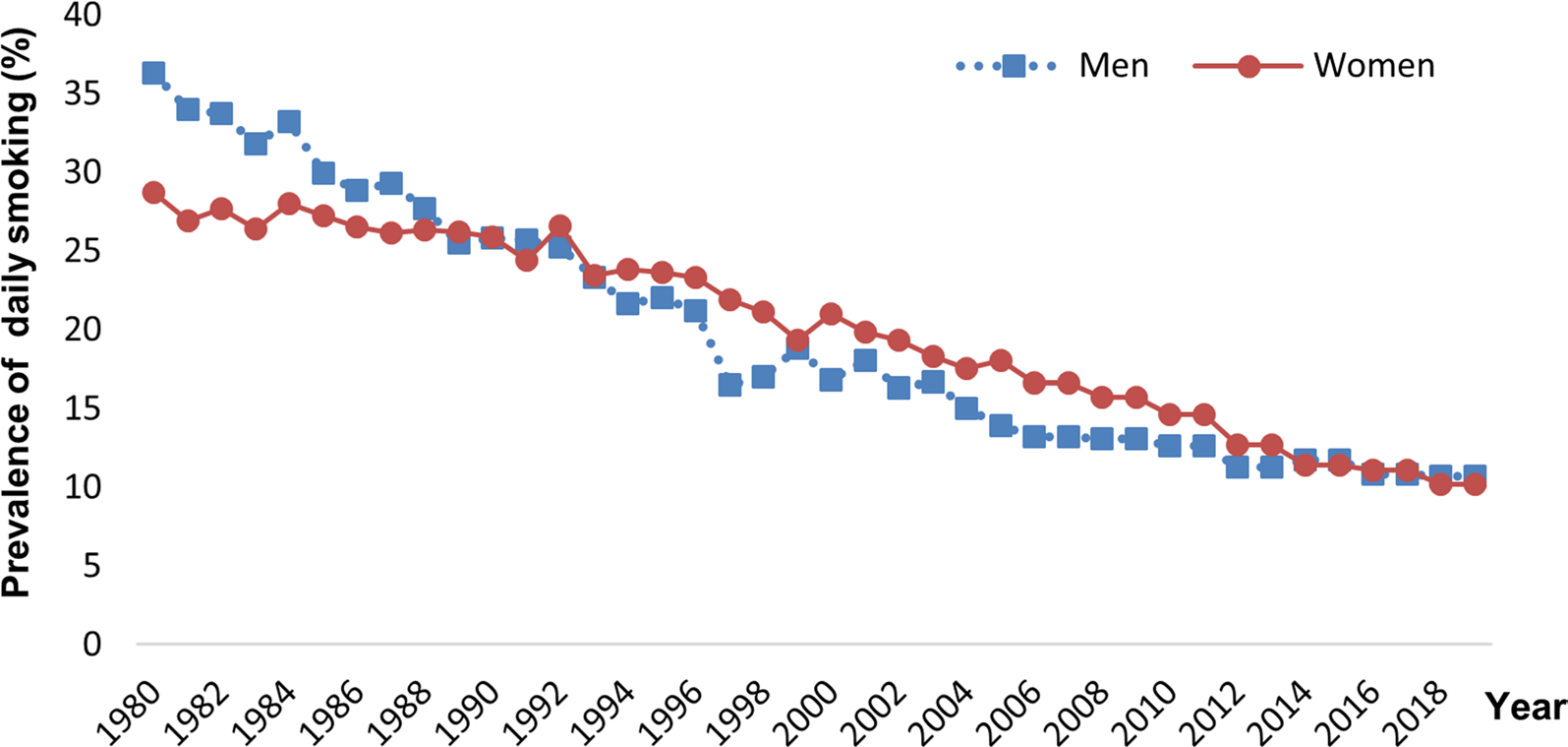
Prevalence of daily smoking in Sweden from 1980 to 2019 in ages 16–84 years by sex, according to Statistics Sweden

The prevalence of COPD was 7.0% in ages 21–78 years in 2009–2012, and the prevalence of moderate to severe (GOLD stage ≥ 2) COPD was 3.5% according to the GOLD [11] fixed ratio definition in combination with respiratory symptoms. When the lower limit of normal definition of COPD was applied, the prevalence estimates were about 30% lower. The prevalence of COPD, particularly of moderate to severe COPD, decreased from 1994 to 2009 and the prevalence in the latter survey is low in comparison with most other studies on COPD prevalence. These results follow a substantial three to fourfold decrease in smoking prevalence in Sweden over 30 years (Fig. 3). Still, COPD was more common among men, and although current smokers were more common among women, the number of packyears and prevalence of occupational exposure was greater among men.

The scientific debate on how to best define COPD is still ongoing [15, 16], which is not surprising as relative to most diseases, COPD is an indeed “young” disease. Most guidelines on COPD rely on the fixed ratio definition of FEV_1_/FVC < 0.70 recommended by GOLD [17]. Clinical physiologists recommend the use of the 5th or 2.5th percentile of the reference value for the FEV_1_/FVC ratio as lower limit of normal (LLN) [18, 19] as also recommended by two ERS task forces [20, 21]. The GOLD strategy document has been revised over the years with nowadays a more pronounced role for patient-reported outcomes, e.g. symptoms and exacerbation history, and not only obstructive spirometry [11].

Differences in criteria for defining COPD together with differences in study designs, age distributions of the studied samples, and regional differences in smoking prevalence and other exposures contribute to different prevalence estimates [20, 22]. Taking all this into account, there are nevertheless possibilities for comparisons, and globally the prevalence estimates of CAO vary from about 10% to 20% among subjects > 40 years of age [23, 24]. The Burden of Obstructive Lung Disease (BOLD) study used identical methods worldwide, and studies following the BOLD protocol have resulted in a prevalence of 15–20% in most European countries in ages > 40 years, and of moderate to severe COPD on average 10%, following the GOLD fixed ratio criterion [25, 26], which is twice as high compared to our results on CAO. However, our results of about 30% lower prevalence based on LLN compared to the fixed ratio definition of COPD are quite in line with the 40% lower prevalence in BOLD [26]. A meta-analysis of European studies estimated the CAO prevalence to be 13.7% in 2010 [27], and recent studies in Canada and within the US NHANES have ended up in similar prevalence [28, 29]. Similar but slightly variable data were reported from Scandinavian countries after the millennial shift, as summarized in Table 5 [3, 8–10, 30–34]. Regarding severity of COPD, several of these studies have found considerably higher prevalence of moderate to severe COPD defined as GOLD grade ≥ 2 than our results, although it should be noted that some of these referred studies are based on pre-bronchodilator spirometry [8, 10, 32].

**Table 5:** Studies from the Scandinavian countries presenting COPD prevalence estimates

Authors [reference]	Country, area	COPD-definition	Study year	Age-span (yy–yy)	Sample size (n)	COPD prevalence based on spirometry according to different criteria			
						Fixed ratio	LLN	Fixed ratio	LLN
						GOLD		GOLD ≥ 2	FEV1 < LLN
Lundbäck et al. [3]	Sweden	FEV_1_/VC	1996–1997	46–77	1237	14.3%	*	8.1%	*
	Norrbotten	Post-BD		(in 3 age groups)					
Kotaniemi et al. [30]	Finland,	FEV_1_/VC	1996–1997	21–70	683	9.4%	*	5.4%	*
	Northern part	Post-BD							
Lindberg et al. [31]	Sweden	FEV_1_/VC	1994	23–72	666	14.1%	*	7.6%	*
	Norrbotten	Pre-BD		46–72		17.1%	*	9.7%	*
Vasankari et al. [8]	Finland	FEV_1_/FVC	1978–1980	30–74	6364	*	M: 4.7%; W: 2.2%	*	M: 3.9%; W: 1.4%
	Nation wide	Pre-BD	2000–2001	30–74	5495	*	M: 4.3%; W: 3.1%	*	M: 3.6%; W: 1.5%
Fabricius et al.^£^, [32]	Denmark	FEV_1_/FVC	2001–2003	≥ 35	5299	17.4%	*	11.2%	*
	Copenhagen	Pre-BD							
Danielsson et al. [33]	Sweden	FEV_1_/FVC	2006–2007	≥ 40	548	16.2%	10.0%	6.7%	*
	Uppsala	Post-BD							
Waatevik et al.^£^, [34]	Norway	FEV_1_/FVC	2003–2005	35–90	1664	13.7%	*	*	*
	Bergen	Post-BD							
Backman et al. [9]	Sweden	FEV_1_/FVC	1994	23–72	660	10.5%	9.3%	8.5%	8.1%
	Norrbotten	Post-BD		46–72	481	13.2%	10.7%	11.6%	9.7%
Backman et al. [9]	Sweden	FEV_1_/FVC	2009	23–72	623	8.5%	6.3%	3.9%	3.2%
	Norrbotten	Post-BD		46–72	465	11.2%	7.4%	5.6%	4.6%
Bhatta et al. [10]	Norway	FEV_1_/FVC	1995–1997	≥ 40	7158	16.7%	10.4%	M: 10.6%; W:6.1%	*
	Nord-Trøndelag	Pre-BD	2006–2008	41–99	8788	14.8%	7.3%	M: 8.2%; W: 6.5%	*

*M* among men, *W* among women, *VC* Highest of slow (SVC) or forced (FVC) expiratory capacity, **£** = Based on follow-up, i.e. not cross-sectional study

In contrast to the large number of cross-sectional surveys studying prevalence of CAO, only few have allowed for studies of trends in prevalence. The Spanish IBERPOC [35] was followed after 10 years by the EPI-SPAN and showed a substantial decrease in the prevalence of CAO GOLD grade ≥ 2 [7], and the Norwegian HUNT study found a decreased prevalence of CAO GOLD grade 2 over eleven years in parallel with decreased smoking prevalence from 29 to 17% [10]. The repeated studies from US NHANES and Finland with follow-up periods of about 20 years found no major prevalence change but a trend towards a decrease in both CAO and smoking [8, 28]. Also based solely on spirometry results, a previous study in the Northern Sweden area revealed a decrease in the prevalence of CAO GOLD grade ≥ 2 over 15 years following a decrease in smoking prevalence from 27 to 16% [9]. Thus, CAO prevalence seems to be decreasing in some high income countries with decades of decreasing smoking prevalence [6, 36].

In line with results from studies in high income countries worldwide [11], smoking remained by far the dominating risk factor for COPD in our study. The magnitude of smoking as risk for developing COPD remained high, but in contrast to our first study on COPD prevalence [3], less than half of elderly smokers had developed COPD. This may be a consequence of both lower numbers of cigarettes smoked per day and a decrease of exposure to environmental tobacco smoke in work places, homes, restaurants, and public places as a result both of large scale society actions at schools and workplaces, in media, and of legislation as well. The proportion of never smokers among subjects with COPD remained low and on similar level as in our previously performed studies [3, 37]. Still COPD was more common among men than women, although the smoking prevalence in Sweden has not been higher in men for decades. Our results indicate that women might have benefited more from the substantial decrease in smoking prevalence, results supported by studies showing that women are more susceptible to the harmful effects of smoking than men [38]. The higher COPD prevalence among men is probably a consequence of the higher number of packyears among smoking men than smoking women along with differences in occupational exposures. Men had a history of more occupational exposure to GDF, which was independently associated with COPD, in line with previous results [12, 39]. Several large industries are located in the study areas, and previous Swedish studies have shown an increased COPD mortality among construction workers [40]. Further, although we did not see independent associations between low socioeconomic status and COPD, it has been shown in previous studies [3, 41, 42]. Thus, it is important that, besides continuing to target smoking rates, public health strategies also aim to reduce the levels of occupational exposures and socioeconomic differences.

As the major part of subjects with CAO but no symptoms were > 60 years of age, adding symptoms to the spirometric definition of chronic airway obstruction, in accordance with recent GOLD recommendations [11], reduces the age-related bias associated with the fixed ratio definition of COPD. As found by others [43, 44], under-diagnosis of COPD was still huge in 2009, although our results show a decreasing trend regarding under-diagnosis. Early recognition of COPD is important, also in younger ages [45, 46], and as it improves, the burden of COPD on the health care system will remain huge despite decreasing prevalence. In line with results from studies by us and others [47, 48], multi-morbidity was common among subjects with COPD, inferring further challenges for care providers. However, a positive result was that the mean age among those with COPD was higher in 2009 than in 1994, implying disease onset at older age and thus improved public health, likely due to successful interventions and legislation targeting smoking.

Overall, the results strongly emphasize the importance of continuous smoking cessation measures, not only among subjects with COPD but among all smokers, along with continuous measures to prevent young people from smoking initiation and political measures to ban smoking from society. Although prevalence decreases, the under-diagnosis of COPD is still substantial. Spirometry is accessible and should be performed in all individuals with respiratory symptoms and/or those extensively exposed to risk factors, in order to enable early intervention and treatment of COPD.

Our study has several strengths, both the OLIN and the WSAS are large studies well reflecting the general population with high participation rates. Studies of non-response have indicated good representativeness of the results for the populations in both the WSAS [49] and OLIN [50] areas, although potential healthy volunteer effects never can be completely ruled out. The study staffs were trained together in order to avoid inter-observer bias, and the same spirometer brand, the Jaeger´s Masterscope, was used in both areas. Furthermore, post-bronchodilator spirometry results were used and quality assurance of the spirometry curves was performed. A further strength is the simultaneous analysis of the fixed ratio and LLN to define CAO. Reassuringly, when including symptoms in the COPD definition, only few subjects were differently classified by LLN. However, there are also weaknesses with our study, e.g. that repeated surveys to enable analyses of time trends only was available from northern Sweden and that there always is a possibility for inter-observer bias when performing structured interviews.

## Conclusion

The prevalence of COPD has decreased in Sweden, and it was considerably lower in 2009–2012, i.e. 7.0%, compared to most previous studies in high income countries including the Scandinavian countries. The prevalence of moderate to severe COPD was particularly low, only 3.5%. The low prevalence follows a three to fourfold decrease in smoking prevalence over the past 30 years. However, smoking still remained by far the most dominating risk factor for COPD.

## Supplementary information

**Additional file 1: Fig. S1**. Study flow chart.

**Additional file 2: Fig. S2**. Distribution of exacerbations and dyspnea among subjects with COPD (FEV1/FVC<0.7 in combination with respiratory symptoms) using a modified GOLD 2020 assessment. Group A) includes n=84 (67%), group B) includes n=8 (8%), group C) includes n=23 (18%) and group D) includes n=11 (9%), while two subjects with COPD lacked information on exacerbations and could not be classified.

**Additional file 3: Tables S1–S6**.

**Additional file 4: Fig. S3**. Interaction analyses for packyears of smoking and exposure to gas, dust or fumes (GDF) and the risk of COPD according to the fixed ratio and LLN-criteria, respectively. Associations are expressed as odds ratios (OR) with 95%CI from logistic regression analyses adjusted for age group and sex. COPD was defined as post-BD FEV1/FVC in combination with respiratory symptoms. N=39 lacked information on either GDF-exposure at work or on packyears of smoking despite being an ever-smoker.

**Additional file 5**. Methods.

## Data Availability

The datasets used and/or analysed during the current study are available from the corresponding author on reasonable request.

## Abbreviations

ANOVA: Analysis of variance
BMI: Body mass index
BOLD: Burden of obstructive lung disease
CAO: Chronic airway obstruction
CI: Confidence interval
COPD: Chronic obstructive pulmonary disease
FEV_1_: Forced expiratory volume in 1 s
FVC: Forced vital capacity
GDF: Gas, dust and fumes
GOLD: Global initiative for chronic obstructive lung disease
LLN: Lower limit of normal
mMRC: Modified Medical Research Council
OLIN: Obstructive lung disease in Northern Sweden
OR: Odds ratio
PR: Prevalence ratio
PY: Packyears
TIA: Transient ischemic attack
WSAS: West Sweden Asthma Study
