# Supplementary figures and images for: Decreased COPD prevalence in Sweden after decades of decrease in smoking
Source: Respir Res. 2020 Oct 28;21:283. doi: 10.1186/s12931-020-01536-4 (PMC7594463; doi:10.1186/s12931-020-01536-4)

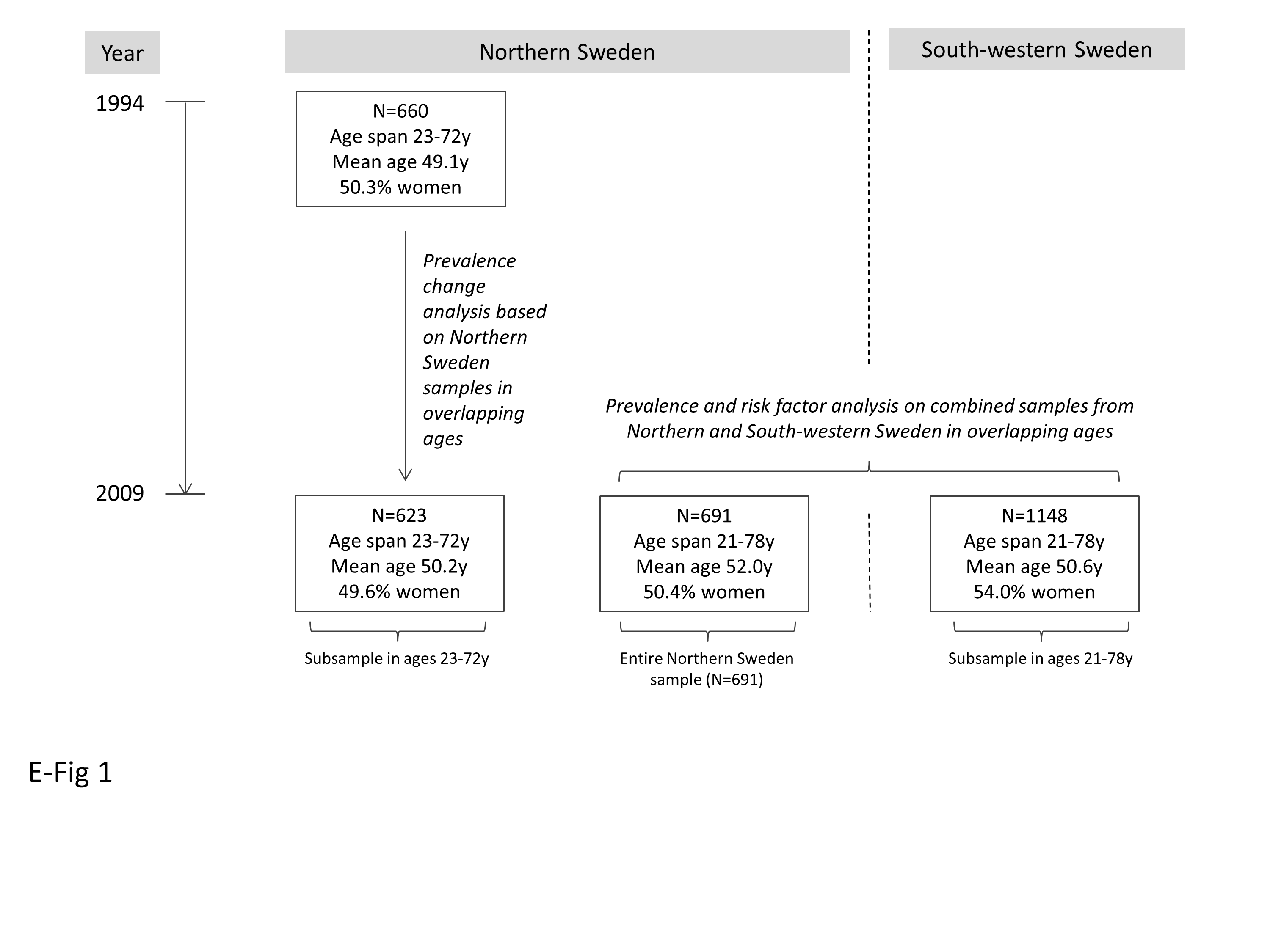

Supplement: Supplementary file 1 — Additional file 1: Fig. S1. Study flow chart. [file 12931_2020_1536_MOESM1_ESM.tif]

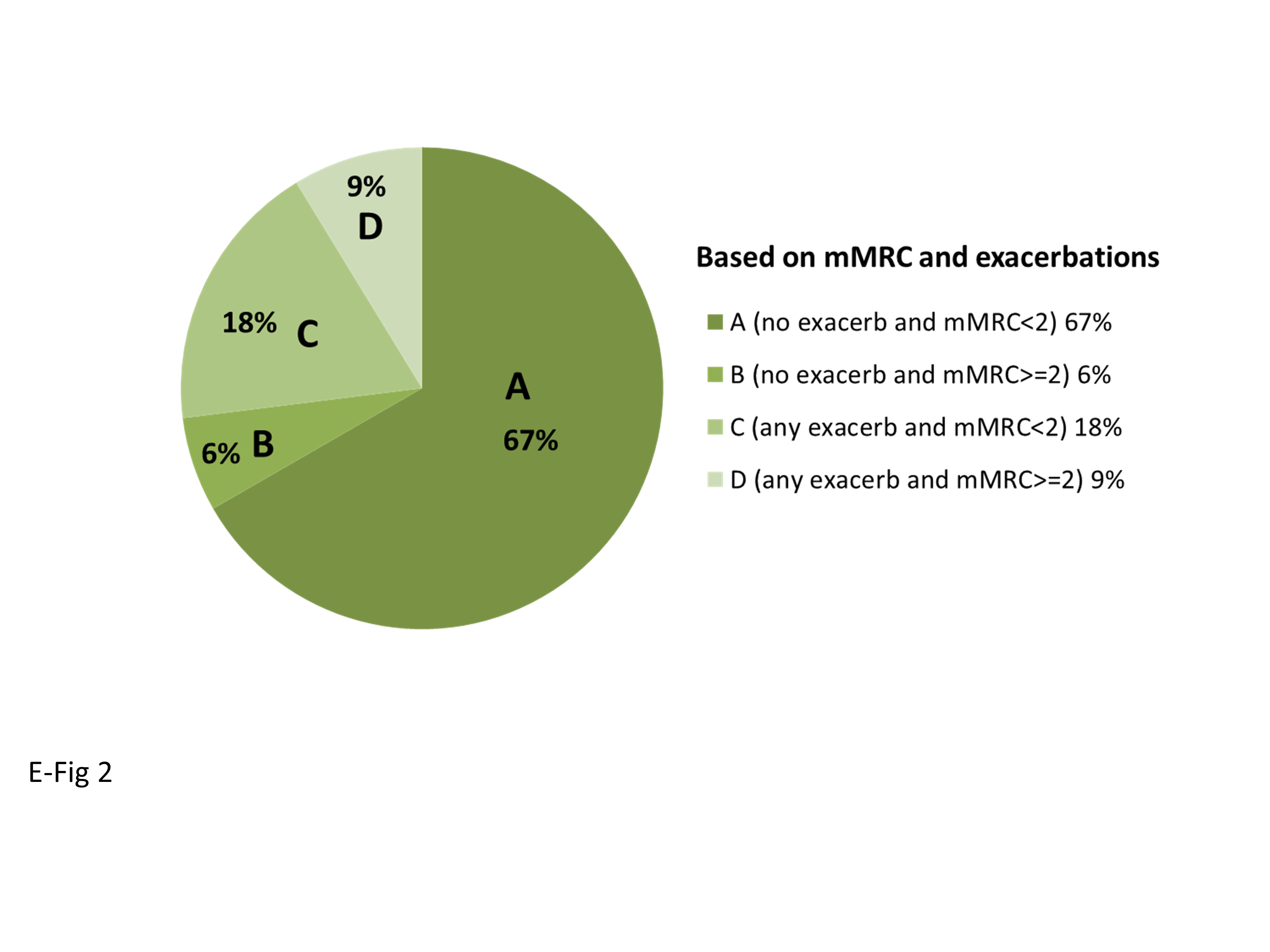

Supplement: Supplementary file 2 — Additional file 2: Fig. S2. Distribution of exacerbations and dyspnea among subjects with COPD (FEV1/FVC<0.7 in combination with respiratory symptoms) using a modified GOLD 2020 assessment. Group A) includes n=84 (67%), group B) includes n=8 (8%), group C) includes n=23 (18%) and group D) includes n=11 (9%), while two subjects with COPD lacked information on exacerbations and could not be classified. [file 12931_2020_1536_MOESM2_ESM.tif]

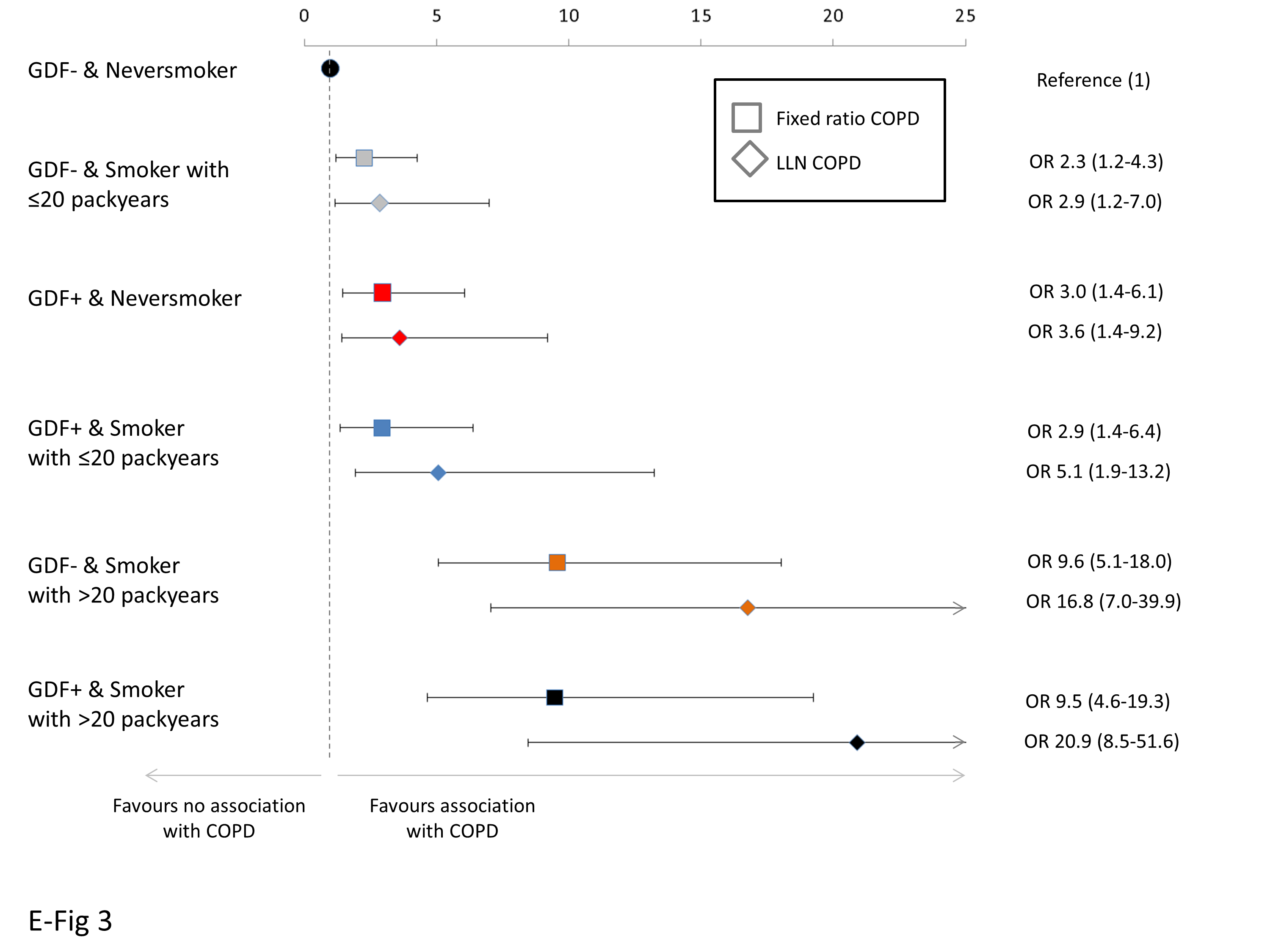

Supplement: Supplementary file 4 — Additional file 4: Fig. S3. Interaction analyses for packyears of smoking and exposure to gas, dust or fumes (GDF) and the risk of COPD according to the fixed ratio and LLN-criteria, respectively. Associations are expressed as odds ratios (OR) with 95%CI from logistic regression analyses adjusted for age group and sex. COPD was defined as post-BD FEV1/FVC in combination with respiratory symptoms. N=39 lacked information on either GDF-exposure at work or on packyears of smoking despite being an ever-smoker. [file 12931_2020_1536_MOESM4_ESM.tif]
