# Supplementary material for: Decreased COPD prevalence in Sweden after decades of decrease in smoking
Source: Respir Res. 2020 Oct 28;21:283. doi: 10.1186/s12931-020-01536-4 (PMC7594463; doi:10.1186/s12931-020-01536-4)
Supplement: Supplementary file 3 — Additional file 3: Tables S1–S6. [file 12931_2020_1536_MOESM3_ESM.docx]

**e-Table 1. Prevalence (%) of comorbidities among subjects with and without COPD**

|  |  | **Non-COPD** | |  | **COPD** | | |  | **Moderate to severe COPD (GOLD≥2)** | | |
| --- | --- | --- | --- | --- | --- | --- | --- | --- | --- | --- | --- |
| **Comorbidity** |  | **n** | **%** |  | **n** | **%** | **P-value** |  | **n** | **%** | **P-value** |
|  |  |  |  |  |  |  |  |  |  |  |  |
| Myocardial infarction |  | 47 | 2.7% |  | 8 | 6.3% | **0.025** |  | 6 | 9.2% | **0.003** |
| Angina pectoris |  | 39 | 2.3% |  | 7 | 5.5% | **0.026** |  | 4 | 6.2% | **0.046** |
| Coronary artery bypass graft |  | 35 | 2.0% |  | 4 | 3.1% | 0.414 |  | 3 | 4.6% | 0.160 |
| Any ischemic heart disease |  | 78 | 4.6% |  | 12 | 9.4% | **0.015** |  | 8 | 12.3% | **0.004** |
|  |  |  |  |  |  |  |  |  |  |  |  |
| Heart failure |  | 6 | 0.4% |  | 4 | 3.1% | **<0.001** |  | 4 | 6.2% | **<0.001** |
| Arrhythmia |  | 86 | 5.0% |  | 8 | 6.3% | 0.544 |  | 5 | 7.7% | 0.339 |
| Any heart disease |  | 200 | 11.7% |  | 25 | 19.5% | **0.009** |  | 14 | 21.5% | **0.017** |
|  |  |  |  |  |  |  |  |  |  |  |  |
| Hypertension (or medicines for hypertension) |  | 418 | 24.4% |  | 56 | 43.8% | **<0.001** |  | 35 | 53.8% | **<0.001** |
| Medication for heart disease |  | 279 | 16.3% |  | 37 | 28.9% | **<0.001** |  | 22 | 33.8% | **<0.001** |
| Medication for heart disease or hypertension |  | 401 | 23.4% |  | 54 | 42.2% | **<0.001** |  | 32 | 49.2% | **<0.001** |
|  |  |  |  |  |  |  |  |  |  |  |  |
| Intermittent claudication |  | 19 | 1.1% |  | 6 | 4.7% | **0.001** |  | 6 | 9.2% | **<0.001** |
| Stroke or TIA |  | 58 | 3.4% |  | 7 | 5.5% | 0.219 |  | 7 | 10.8% | **0.002** |
| Diabetes |  | 72 | 4.2% |  | 11 | 8.6% | **0.021** |  | 7 | 10.8% | **0.012** |
| Rheumatic disease |  | 90 | 5.3% |  | 7 | 5.5% | 0.919 |  | 3 | 4.6% | 0.819 |
|  |  |  |  |  |  |  |  |  |  |  |  |
| At least one comorbidity (listed above) |  | 587 | 34.3% |  | 66 | 51.6% | **<0.001** |  | 38 | 58.5% | **<0.001** |
| Number of comorbidities ≥2 |  | 178 | 10.4% |  | 29 | 22.7% | **<0.001** |  | 21 | 32.3% | **<0.001** |
| Number of comorbidities ≥3 |  | 65 | 3.8% |  | 14 | 10.9% | **<0.001** |  | 12 | 18.5% | **<0.001** |
| Number of comorbidities ≥4 |  | 30 | 1.8% |  | 5 | 3.9% | 0.086 |  | 5 | 7.7% | **0.001** |

COPD=post-bronchodilator FEV1/FVC<0.7 in combination with respiratory symptoms. Moderate to severe COPD=COPD with FEV1<80% of predicted. Ischemic heart disease: Any of myocardial infarction, coronary artery bypass graft, or angina pectoris. Any heart disease: Ischemic heart disease, arrhythmia or other (unspecified) heart disease. TIA=transient ischemic attack. P-values from Chi-square test compared to Non-COPD. Bold font indicates p<0.05.

**e-Table 2.** **Risk factors for chronic airway obstruction (CAO) and COPD based on the Lower Limit of Normal definition. Results presented as Odds Ratios (OR) with 95% Confidence Intervals (CI) from multiple logistic regression analyses.**

|  |  |  | CAO |  |  |  |  |  |  |  | COPD |  |  |  |
| --- | --- | --- | --- | --- | --- | --- | --- | --- | --- | --- | --- | --- | --- | --- |
|  | FEV1/FVC<LLN | | | | | |  |  | FEV1/FVC<LLN | | | | | |
| Covariate | OR |  | 95%CI | | |  |  |  | OR |  | 95%CI | | |  |
|  |  |  |  |  |  |  |  |  |  |  |  |  |  |  |
| <40y | Reference | | |  |  |  |  |  | Reference | | |  |  |  |
| 40-60y | 0.74 | ( | 0.39 | - | 1.42 | ) | |  | 0.83 | ( | 0.39 | - | 1.77 | ) |
| >60y | 1.00 | ( | 0.52 | - | 1.95 | ) | |  | 0.92 | ( | 0.42 | - | 2.02 | ) |
|  |  |  |  |  |  |  |  |  |  |  |  |  |  |  |
| Male sex | **1.67** | **(** | **1.05** | **-** | **2.66** | **)** | |  | **1.74** | **(** | **1.04** | **-** | **2.92** | **)** |
|  |  |  |  |  |  |  |  |  |  |  |  |  |  |  |
| University education | Reference | | |  |  |  |  |  | Reference | | |  |  |  |
| Non-university edu | 1.25 | ( | 0.76 | - | 2.04 | ) | |  | 1.15 | ( | 0.67 | - | 1.99 | ) |
|  |  |  |  |  |  |  |  |  |  |  |  |  |  |  |
| Never-smoker | Reference | | |  |  |  |  |  | Reference | | |  |  |  |
| ≤10 packyears | 1.43 | ( | 0.77 | - | 2.68 | ) | |  | 1.52 | ( | 0.72 | - | 3.20 | ) |
| 11-20 packyears | **2.57** | **(** | **1.24** | **-** | **5.36** | **)** | |  | **3.63** | **(** | **1.61** | **-** | **8.14** | **)** |
| 21-30 packyears | **4.87** | **(** | **2.41** | **-** | **9.83** | **)** | |  | **7.53** | **(** | **3.49** | **-** | **16.23** | **)** |
| >30 packyears | **9.60** | **(** | **5.08** | **-** | **18.14** | **)** | |  | **13.69** | **(** | **6.70** | **-** | **27.98** | **)** |
|  |  |  |  |  |  |  |  |  |  |  |  |  |  |  |
| Exp to GDF at work | **1.64** | **(** | **1.04** | **-** | **2.58** | **)** | |  | **1.83** | **(** | **1.11** | **-** | **3.00** | **)** |
|  |  |  |  |  |  |  |  |  |  |  |  |  |  |  |

Binomial logistic regression for COPD vs non-COPD and CAO vs non-CAO, respectively.
CAO = Post-bronchodilator chronic airway obstruction according to the LLN-definition (FEV_1_/FVC<LLN).
COPD = CAO in combination with respiratory symptoms. GDF=Gas, dust or fumes.
LLN based on the OLIN reference values. Bold font indicates p<0.05.

**e-Table 3. Characteristics and prevalence of COPD and moderate to severe COPD (GOLD>=2) in the Northern Sweden sample in 1994.**

|  |  | ≤40 years | | | | | | |  | >40 years | | | | | | | | |  |  | | |  | |  |
| --- | --- | --- | --- | --- | --- | --- | --- | --- | --- | --- | --- | --- | --- | --- | --- | --- | --- | --- | --- | --- | --- | --- | --- | --- | --- |
|  |  | Women  (n=98) | |  | Men  (n=81) | | | |  | Women  (n=234) | | | |  | Men  (n=247) | | | |  | All subjects  (n=660, age 23-72y) | | | | |  |
|  |  |  |  | | |  |  |  | | |  |  |  | | |  |  |  | | |  |  | |  | |
| Age | mean (SD) | 32.8 | (5.0) | | |  | 33.3 | (4.6) | | |  | 55.1 | (9.0) | | |  | 55.0 | (8.6) | | |  | 49.1 | | (12.6) | |
| BMI | mean (SD) | 24.3 | (3.7) | | |  | 25.6 | (3.1) | | |  | 25.8 | (4.1) | | |  | 26.1 | (3.0) | | |  | 25.7 | | (3.6) | |
|  |  |  |  | | |  |  |  | | |  |  |  | | |  |  |  | | |  |  | |  | |
| Non-smokers | n (%) | 49 | (50.5%) | | |  | 46 | (56.8%) | | |  | 115 | (49.1%) | | |  | 86 | (34.8%) | | |  | 296 | | (44.9%) | |
| Ex-smokers | n (%) | 22 | (22.7%) | | |  | 17 | (21.0%) | | |  | 44 | (18.8%) | | |  | 105 | (42.5%) | | |  | 188 | | (28.5%) | |
| Current smokers | n (%) | 26 | (26.8%) | | |  | 18 | (22.2%) | | |  | 75 | (32.1%) | | |  | 56 | (22.7%) | | |  | 175 | | (26.6%) | |
|  |  |  |  | | |  |  |  | | |  |  |  | | |  |  |  | | |  |  | |  | |
| COPD | n (%) | 4 | (4.1%) | | |  | 5 | (6.2%) | | |  | 24 | (10.3%) | | |  | 28 | (11.3%) | | |  | 61 | | (9.2%) | |
| GOLD≥2 | n (%) | 1 | (1.0%) | | |  | 5 | (6.2%) | | |  | 20 | (8.5%) | | |  | 27 | (10.9%) | | |  | 53 | | (8.0%) | |
|  |  |  |  | | |  |  |  | | |  |  |  | | |  |  |  | | |  |  | |  | |

One man >40 years of age lacks data on smoking habits. COPD=post-bronchodilator FEV1/FVC<0.7 in combination with respiratory symptoms. GOLD≥2 = moderate to severe COPD =COPD with FEV1<80% of predicted.

**e-Table 4. Prevalence (%) of chronic airway obstruction (CAO) and COPD, respectively, using the Lower Limit of Normal (LLN) criterion based on the GLI reference values, by age group, smoking habits and among all. Prevalence ratios (PR) with 95% CI from unadjusted Poisson regression analyses comparing prevalence among all in 2009 with 1994.**

|  |  |  | Age groups | | | | |  | Smoking habits | | |  |  |  | Prevalence Ratio | |
| --- | --- | --- | --- | --- | --- | --- | --- | --- | --- | --- | --- | --- | --- | --- | --- | --- |
|  | Year |  | <40y |  | 41-60y |  | >60y |  | Non-smokers | Ex-smokers | Current smokers |  | All |  | PR | 95%CI |
|  | 1994 |  | 8.4% |  | 8.9% |  | 11.1% |  | 4.1% | 8.5% | 18.9% |  | 9.3% |  |  |  |
| CAO | 2009 |  | 3.8% |  | 5.1% |  | 9.2% |  | 2.3% | 5.1% | 18.0% |  | 5.8%* |  | 0.63 | (0.42-0.93) |
|  |  |  |  |  |  |  |  |  |  |  |  |  |  |  |  |  |
|  | 1994 |  | 5.6% |  | 7.4% |  | 9.7% |  | 2.4% | 6.4% | 17.1% |  | 7.4% |  |  |  |
| COPD | 2009 |  | 0.6%* |  | 3.8% |  | 5.9% |  | 0.7% | 3.3% | 13.0% |  | 3.5%* |  | 0.48 | (0.29-0.78) |
|  |  |  |  |  |  |  |  |  |  |  |  |  |  |  |  |  |
| *P<0.05 compared to in 1994 | |  |  |  |  |  |  |  |  |  |  |  |  |  |  |  |
| Based on post-BD spirometry and GLI reference values | | | | | |  |  |  |  |  |  |  |  |  |  |  |
| Chronic airway obstruction=based only on spirometry (FEV1/FVC<LLN) | | | | | | | |  |  |  |  |  |  |  |  |  |
| COPD=Chronic airway obstruction in combination with respiratory symptoms | | | | | | | | | |  |  |  |  |  |  |  |

**e-Table 5. Risk factors for chronic airway obstruction (CAO) and COPD according to the fixed ratio and LLN criteria, respectively. Results presented as Odds Ratios (OR) with 95% Confidence Intervals (CI) from crude (unadjusted) logistic regression analyses.**

|  |  | Fixed ratio criterion (FEV_1_/FVC<0.7) | | | | | | | | | | | |  |  | Lower Limit of Normal criterion (FEV_1_/FVC<LLN) | | | | | | | | | | | | | |
| --- | --- | --- | --- | --- | --- | --- | --- | --- | --- | --- | --- | --- | --- | --- | --- | --- | --- | --- | --- | --- | --- | --- | --- | --- | --- | --- | --- | --- | --- |
|  |  |  |  | CAO |  |  |  |  |  |  | COPD |  |  |  |  |  |  | CAO |  |  |  |  |  |  |  | COPD |  |  |  |
| Covariate |  | OR |  | (95%CI) | | |  |  | OR |  | (95%CI) | | |  |  | OR |  | (95%CI) | | |  |  |  | OR |  | (95%CI) | | |  |
|  |  |  |  |  |  |  |  |  |  |  |  |  |  |  |  |  |  |  |  |  |  |  |  |  |  |  |  |  |  |
| Age <40y |  | Reference |  |  |  |  |  |  | Reference |  |  |  |  |  |  | Reference |  |  |  |  |  |  |  | Reference |  |  |  |  |  |
| Age 40-60y |  | **2.26** | **(** | **1.21** | **-** | **4.21** | **)** | | **2.57** | **(** | **1.28** | **-** | **5.17** | **)** | | 1.31 | ( | 0.73 | - | 2.34 | ) | |  | 1.72 | ( | 0.88 | - | 3.34 | ) |
| Age >60y |  | **6.89** | **(** | **3.81** | **-** | **12.46** | **)** | | **6.59** | **(** | **3.37** | **-** | **12.90** | **)** | | **2.37** | **(** | **1.35** | **-** | **4.18** | **)** | |  | **2.73** | **(** | **1.41** | **-** | **5.26** | **)** |
|  |  |  |  |  |  |  |  |  |  |  |  |  |  |  |  |  |  |  |  |  |  |  |  |  |  |  |  |  |  |
| Male sex |  | 1.36 | ( | 0.98 | - | 1.88 | ) | | **1.47** | **(** | **1.02** | **-** | **2.11** | **)** | | **2.40** | **(** | **1.58** | **-** | **3.66** | **)** | |  | **2.58** | **(** | **1.62** | **-** | **4.10** | **)** |
|  |  |  |  |  |  |  |  |  |  |  |  |  |  |  |  |  |  |  |  |  |  |  |  |  |  |  |  |  |  |
| University education |  | Reference |  |  |  |  |  |  | Reference |  |  |  |  |  |  | Reference |  |  |  |  |  |  |  | Reference |  |  |  |  |  |
| Non-university education |  | **1.79** | **(** | **1.25** | **-** | **2.56** | **)** | | **1.76** | **(** | **1.19** | **-** | **2.62** | **)** | | **2.04** | **(** | **1.30** | **-** | **3.20** | **)** | |  | **2.09** | **(** | **1.27** | **-** | **3.43** | **)** |
|  |  |  |  |  |  |  |  |  |  |  |  |  |  |  |  |  |  |  |  |  |  |  |  |  |  |  |  |  |  |
| Never-smoker |  | Reference |  |  |  |  |  |  | Reference |  |  |  |  |  |  | Reference |  |  |  |  |  |  |  | Reference |  |  |  |  |  |
| ≤10 packyears |  | 1.20 | ( | 0.73 | - | 1.97 | ) | | 1.37 | ( | 0.78 | - | 2.41 | ) | | 1.45 | ( | 0.79 | - | 2.66 | ) | |  | 1.54 | ( | 0.75 | - | 3.15 | ) |
| 11-20 packyears |  | **2.74** | **(** | **1.62** | **-** | **4.64** | **)** | | **3.01** | **(** | **1.66** | **-** | **5.47** | **)** | | **2.30** | **(** | **1.15** | **-** | **4.60** | **)** | |  | **3.12** | **(** | **1.46** | **-** | **6.66** | **)** |
| 21-30 packyears |  | **5.39** | **(** | **3.21** | **-** | **9.03** | **)** | | **7.07** | **(** | **4.04** | **-** | **12.36** | **)** | | **5.64** | **(** | **3.02** | **-** | **10.53** | **)** | |  | **8.38** | **(** | **4.27** | **-** | **16.5** | **)** |
| >30 packyears |  | **9.91** | **(** | **6.08** | **-** | **16.14** | **)** | | **11.17** | **(** | **6.53** | **-** | **19.10** | **)** | | **11.04** | **(** | **6.25** | **-** | **19.49** | **)** | |  | **14.90** | **(** | **7.93** | **-** | **28.0** | **)** |
|  |  |  |  |  |  |  |  |  |  |  |  |  |  |  |  |  |  |  |  |  |  |  |  |  |  |  |  |  |  |
| Exp to GDF at work |  | **1.61** | **(** | **1.14** | **-** | **2.26** | **)** | | **1.84** | **(** | **1.27** | **-** | **2.67** | **)** | | **2.31** | **(** | **1.54** | **-** | **3.47** | **)** | |  | **2.62** | **(** | **1.69** | **-** | **4.06** | **)** |
|  |  |  |  |  |  |  |  |  |  |  |  |  |  |  |  |  |  |  |  |  |  |  |  |  |  |  |  |  |  |
| Family history of OAD |  | 1.05 | ( | 0.72 | - | 1.54 | ) | | 1.15 | ( | 0.76 | - | 1.74 | ) | | 1.41 | ( | 0.91 | - | 2.18 | ) | |  | 1.58 | ( | 0.99 | - | 2.52 | ) |
|  |  |  |  |  |  |  |  |  |  |  |  |  |  |  |  |  |  |  |  |  |  |  |  |  |  |  |  |  |  |

Binomial logistic regression for COPD vs non-COPD and CAO vs non-CAO, respectively, according to the respective criteria. CAO = Post-bronchodilator chronic airway obstruction according to the specified definition (FEV_1_/FVC<0.7 or FEV_1_/FVC<LLN). COPD = CAO in combination with respiratory symptoms. OAD=Obstructive airway diseases (asthma or chronic bronchitis). LLN based on the OLIN reference values for spirometry. Bold font indicates p<0.05.

**e-Table 6. Prevalence of respiratory symptoms during the last 12 months among subjects with and without COPD according to the fixed ratio and LLN criteria, respectively.**

|  | Fixed ratio (FEV_1_/FVC<0.7) | | | | | |  | LLN (FEV_1_/FVC<LLN) | | | | | |
| --- | --- | --- | --- | --- | --- | --- | --- | --- | --- | --- | --- | --- | --- |
|  | COPD | |  | Non-COPD | |  |  | COPD | |  | Non-COPD | |  |
|  | n | % |  | n | % | p-value |  | n | % |  | n | % | p-value |
|  |  |  |  |  |  |  |  |  |  |  |  |  |  |
| Longstanding cough | 41 | 32.0% |  | 296 | 17.3% | <0.001 |  | 28 | 32.2% |  | 309 | 17.6% | 0.001 |
| Sputum production | 92 | 71.9% |  | 594 | 34.7% | <0.001 |  | 61 | 70.1% |  | 625 | 35.7% | <0.001 |
| Chronic productive cough | 36 | 28.1% |  | 187 | 10.9% | <0.001 |  | 20 | 23.0% |  | 203 | 11.6% | 0.001 |
| mMRC Dyspnea scale grade ≥1 | 85 | 67.5% |  | 713 | 42.2% | <0.001 |  | 63 | 74.1% |  | 735 | 42.5% | <0.001 |
| Recurrent wheeze | 62 | 48.4% |  | 222 | 13.0% | <0.001 |  | 46 | 52.9% |  | 238 | 13.6% | <0.001 |
| Persistent wheeze | 15 | 11.7% |  | 34 | 2.0% | <0.001 |  | 9 | 10.3% |  | 40 | 2.3% | <0.001 |
| Attacks of shortness of breath | 22 | 17.2% |  | 177 | 10.3% | 0.016 |  | 19 | 21.8% |  | 180 | 10.3% | 0.001 |
|  |  |  |  |  |  |  |  |  |  |  |  |  |  |
| Any of the above | 128 | 100.0% |  | 1125 | 65.8% | <0.001 |  | 87 | 100.0% |  | 1166 | 66.6% | <0.001 |
|  |  |  |  |  |  |  |  |  |  |  |  |  |  |

mMRC = modified Medical Research Council (range 0-4). COPD=Post-bronchodilator chronic airway obstruction according to the specified definition (FEV_1_/FVC<0.7 or FEV_1_/FVC<LLN) in combination with any of the respiratory symptoms listed above. LLN based on the OLIN reference values for spirometry. P-values from Chi-square test comparing COPD and Non-COPD. Bold font indicates p<0.05.
