# Supplementary material for: Decreased COPD prevalence in Sweden after decades of decrease in smoking
Source: Respir Res. 2020 Oct 28;21:283. doi: 10.1186/s12931-020-01536-4 (PMC7594463; doi:10.1186/s12931-020-01536-4)
Supplement: Supplementary file 5 — Additional file 5. Methods. [file 12931_2020_1536_MOESM5_ESM.docx]

**On-line supplement**

**Methods - additional information**

*Study areas*

In south-western Sweden, the region of West Gothia (Västra Götaland) includes rural areas, small and medium sized towns, and the second city of Sweden, Gothenburg. The population exceeds 1,600,000 in an area of 24,000 km^2.^ The climate is Atlantic. In northern Sweden, the northernmost Swedish region, Norrbotten, comprises 25% of the area of Sweden, but is sparsely inhabited with a population of less than 300,000, and the climate is subarctic. Almost 20% of the population of Sweden resides in the two areas.

*Study population*

In south-western Sweden a questionnaire was distributed in 2008 to 30,000 randomly selected subject aged 16-75 years. These 30,000 individuals were randomly selected from the Swedish population register. The response rate was 62% of those who could be traced[1]. A study of non-response demonstrated high representativeness for the population in the region[2]. Of the responders, 2,000 subjects were randomly selected and invited to clinical examinations including pre- and post-bronchodilator spirometry and structured interviews, and 1,158 subjects (58%) participated in 2009-2012 with technique of acceptable quality[3,4].

In Norrbotten, two randomly selected samples of the population in ages 20-69 years and 30-84 years, respectively, were invited to participate in a postal questionnaire survey in 2006. Both samples have previously been described in detail[5,6]. These samples were initially randomly selected from the Swedish population register within the specified age spans. Overall, 12,055 subjects (80% of the invited) participated. After stratification by the age and sex distribution of the population in order to reflect the population of the region, a randomly selected sample of the questionnaire responders, n=1,016, was invited to clinical examinations in 2009 including structured interviews and pre- and post- bronchodilator spirometry. Examinations with adequate technique were performed by 726 (71.5%)[7] and the representativeness of the participants for the population in the region was good[8].

Data have been pooled from the clinical examinations in the two areas in the age-range of 21-78 years (n=1,839), which overlapped both centers.

*Questionnaire*

The same questionnaire was used in both studies; the Swedish OLIN-questionnaire[9,10], which has been used in several national and international epidemiological studies[1,10-13], and validated against the Global Allergy and Asthma European Network (GA^2^LEN) questionnaire[14]. It consists of a self-administrated short version and a version for interviews with additional questions from the GA^2^LEN questionnaire[15]. The questions are focused on respiratory symptoms and diseases, their comorbidities, medication, family history of obstructive airway diseases and allergy, smoking habits, occupation, socio-economic status, area of domicile, and other potential risk factors for respiratory diseases. The interview questions regarding smoking provided detailed information enabling calculation of smoking habits both in terms of being a non-smoker, ex-smoker (quit since at least one year) or current smoker, as well as the calculation of packyears of smoking among ex- and current smokers.

*Spirometry*

A Masterscope (Jaeger) spirometer, with daily check-ups, was used in both study areas. At least three slow vital capacity (SVC) maneuvers were performed followed by forced vital capacity (FVC) measurements at least three and maximum six times. In south-western Sweden, the difference between the two best FVC and the two best forced expiratory volume during the first second of the expiration (FEV_1_), respectively, had to be <5% and <150 ml, or <100 ml for values <2.0 liters, and the bronchodilation test was performed in all subjects using a combination of 0.4 mg salbutamol and 80 mcg ipratropium bromide via spacer. In northern Sweden, the difference between the two best FVC and the two best FEV_1_, respectively, had to be <5%, or <100 ml for values <2.0 liters, and the bronchodilation test was performed in all subjects using 0.4 mg salbutamol via discus. Post-bronchodilator spirometric values were defined as the highest values before or after reversibility testing. Height and weight was measured at the examination, and age was calculated as the difference between date of birth and date of examination. The internally and externally validated OLIN reference values for spirometry were used as they fit well in both areas as they reflect the healthy general population better than the Global Lung Initiative (GLI) reference values[16,17]. In sensitivity analyses the GLI reference values[18] have been used.

*Prevalence change from 1994 to 2009 in Northern Sweden*

In order to study prevalence change, the Northern Sweden (county of Norrbotten) results from 2009 were compared with results from a study performed in 1994 in the same area of Northern Sweden as the present study and in the same age-span, as previously described[7,19]. The interview questionnaire used in 1994 included the same validated questions as in 2009, although some additional questions had been added in 2009. In 1994, 660 subjects (68.0% of invited, in ages 23-72 years which overlapped the 2009 age-span) participated at clinical examinations including an almost identical structured interview as in 2009. The spirometry procedure followed the ERS/ATS guidelines[20], but with a repeatability criterion of <5%, or <100 ml in case the values were <2.0 liters, difference between the two best FVC and the two best FEV_1_ values, respectively. A dry volume spirometer, Minjhard Vicatest 5, was used with daily check-ups of the calibration. Reversibility testing was performed using 0.4 mg salbutamol via discus in all subjects with FEV_1_ <90% of predicted or a ratio of FEV_1_/VC<0.7, where VC was defined as the highest of forced or slow expiratory volume. Post-bronchodilator spirometric values were defined as the highest values before or after reversibility testing. Height and weight was measured at the examination, and age was calculated as the difference between date of birth and date of examination. All subjects with both post-bronchodilator FEV_1_/FVC<0.7 and respiratory symptoms in the two surveys also had pre-bronchodilator FEV_1_/VC<0.7 why the different selection procedures for invitation to bronchodilator testing in 1994 compared to in 2009 did not affect the results. In the comparison of results on prevalence change between 1994 and 2009, the COPD definition was the following: post-bronchodilator FEV1/FVC<0.7 in combination with at least one of the following respiratory symptoms: longstanding cough, chronic productive cough, mMRC dyspnea scale ≥2, recurrent wheeze, persistent wheeze and/or attacks of shortness of breath, all within the last 12 months. Results based on the lower limit of normal (LLN) definition of chronic airway obstruction was calculated to enable comparisons with other studies using this definition, which is elaborated upon in the below section.

*The lower limit of normal (LLN) criterion*

The LLN was defined as the 5^th^ percentile of the reference value, in line with recommendations by two ERS Task Forces[18,21].

In line with the fixed ratio definition in the main text, CAO was defined as post-bronchodilator FEV1/FVC<LLN, and COPD was defined as CAO in combination with chronic or recurrent respiratory symptoms. Based on the FEV1/FVC<LLN definition, the prevalence of CAO and COPD was 5.7% (95%CI 4.6-6.7) and 4.7% (95%CI 3.8-5.7) in 2009-2012. When limiting the sample to ages ≥40years, the corresponding prevalence was 6.3% (95%CI 5.0-7.6) and 5.4% (95%CI 4.2-6.6). The significant decrease in both CAO and COPD from 1994 to 2009 in Northern Sweden shown by the fixed ratio criterion was confirmed by the analyses based on the LLN criterion, with a decrease in CAO of 35.8% (95%CI 6.4-56.0) and in COPD of 42.0% (95%CI 10.1-62.6). The decrease was thus significant when based on the Swedish reference values for spirometry, and also when using the GLI reference values, as presented in on-line Table 4. Adjusted risk factor analyses for the LLN definition of COPD revealed similar results as for the fixed ratio definition of COPD with two exceptions. Age was not a significant risk factor for CAO or COPD according to the LLN-definition, while male sex was a significant risk factor with OR 1.7 (95%CI 1.05-2.7) for CAO and OR 1.7 (95%CI 1.04-2.9) for COPD, and the OR:s for smoking were higher than for the fixed ratio-definition (on-line Table 5).

References

(1) Lotvall J, Ekerljung L, Ronmark EP, Wennergren G, Linden A, Ronmark E, et al. West Sweden Asthma Study: prevalence trends over the last 18 years argues no recent increase in asthma. Respir Res 2009 Oct 12;10:94.

(2) Ronmark EP, Ekerljung L, Lotvall J, Toren K, Ronmark E, Lundback B. Large scale questionnaire survey on respiratory health in Sweden: effects of late- and non-response. Respir Med 2009 Dec;103(12):1807-1815.

(3) Ekerljung L, Bjerg A, Bossios A, Axelsson M, Toren K, Wennergren G, et al. Five-fold increase in use of inhaled corticosteroids over 18 years in the general adult population in west Sweden. Respir Med 2014 May;108(5):685-693.

(4) Nwaru BI, Ekerljung L, Rådinger M, Bjerg A, Mincheva R, Malmhäll C, Axelsson M, Wennergren G, Lotvall J, Lundbäck B. Cohort profile: the West Sweden Asthma Study (WSAS): a multidisciplinary population-based longitudinal study of asthma, allergy and respiratory conditions in adults. BMJ Open. 2019 Jun 19;9(6):e027808.

(5) Lindstrom M, Kotaniemi J, Jonsson E, Lundback B. Smoking, respiratory symptoms, and diseases : a comparative study between northern Sweden and northern Finland: report from the FinEsS study. Chest 2001 Mar;119(3):852-861.

(6) Backman H, Hedman L, Jansson SA, Lindberg A, Lundback B, Ronmark E. Prevalence trends in respiratory symptoms and asthma in relation to smoking - two cross-sectional studies ten years apart among adults in northern Sweden. World Allergy Organ J 2014 Jan 2;7(1):1-4551-7-1.

(7) Backman H, Eriksson B, Rönmark E, Hedman L, Stridsman C, Jansson S, et al. Decreased prevalence of moderate to severe COPD over 15 years in northern Sweden. Respir Med 2016 22/3;114:103-110.

(8) Warm K, Lindberg A, Lundback B, Ronmark E. Increase in sensitization to common airborne allergens among adults - two population-based studies 15 years apart. Allergy Asthma Clin Immunol 2013 Jun 11;9(1):20-1492-9-20. eCollection 2013.

(9) Lundback B, Nystrom L, Rosenhall L, Stjernberg N. Obstructive lung disease in northern Sweden: respiratory symptoms assessed in a postal survey. Eur Respir J 1991 Mar;4(3):257-266.

(10) Pallasaho P, Lundback B, Laspa SL, Jonsson E, Kotaniemi J, Sovijarvi AR, et al. Increasing prevalence of asthma but not of chronic bronchitis in Finland? Report from the FinEsS-Helsinki Study. Respir Med 1999 Nov;93(11):798-809.

(11) Kotaniemi JT, Lundback B, Nieminen MM, Sovijarvi AR, Laitinen LA. Increase of asthma in adults in northern Finland?--a report from the FinEsS study. Allergy 2001 Feb;56(2):169-174.

(12) Raukas-Kivioja A, Raukas E, Loit HM, Kiviloog J, Ronmark E, Larsson K, et al. Allergic sensitization among adults in Tallinn, Estonia. Clin Exp Allergy 2003 Oct;33(10):1342-1348.

(13) Lam HT, Ekerljung L, T Formula See Text Ng,N.F., Ronmark E, Larsson K, Lundback B. Prevalence of COPD by disease severity in men and women in northern Vietnam. COPD 2014 Sep;11(5):575-581.

(14) Ekerljung L, Ronmark E, Lotvall J, Wennergren G, Toren K, Lundback B. Questionnaire layout and wording influence prevalence and risk estimates of respiratory symptoms in a population cohort. Clin Respir J 2013 Jan;7(1):53-63.

(15) Bousquet J, Burney PG, Zuberbier T, Cauwenberge PV, Akdis CA, Bindslev-Jensen C, et al. GA2LEN (Global Allergy and Asthma European Network) addresses the allergy and asthma 'epidemic'. Allergy 2009 Jul;64(7):969-977.

(16) Backman H, Lindberg A, Sovijarvi A, Larsson K, Lundback B, Ronmark E. Evaluation of the global lung function initiative 2012 reference values for spirometry in a Swedish population sample. BMC Pulm Med 2015 Mar 25;15:26-015-0022-2.

(17) Backman H, Lindberg A, Oden A, Ekerljung L, Hedman L, Kainu A, et al. Reference values for spirometry - report from the Obstructive Lung Disease in Northern Sweden studies. Eur Clin Respir J 2015 Jul 20;2:10.3402/ecrj.v2.26375. eCollection 2015.

(18) Quanjer PH, Stanojevic S, Cole TJ, Baur X, Hall GL, Culver BH, et al. Multi-ethnic reference values for spirometry for the 3-95-yr age range: the global lung function 2012 equations. Eur Respir J 2012 Dec;40(6):1324-1343.

(19) Lindberg A, Jonsson AC, Ronmark E, Lundgren R, Larsson LG, Lundback B. Prevalence of chronic obstructive pulmonary disease according to BTS, ERS, GOLD and ATS criteria in relation to doctor's diagnosis, symptoms, age, gender, and smoking habits. Respiration 2005 Sep-Oct;72(5):471-479.

(20) Miller MR, Hankinson J, Brusasco V, Burgos F, Casaburi R, Coates A, et al. Standardisation of spirometry. Eur Respir J 2005 Aug;26(2):319-338.

(21) Bakke PS, Ronmark E, Eagan T, Pistelli F, Annesi-Maesaon I, Maly M, Meren M, Vermeire Dagger P, Vestbo J, Viegi G, Zielinski J, Lundbäck B; European Respiratory Society Task Force. Recommendations for epidemiological studies on COPD. Eur Respir J. 2011;38(6):1261-1277.
